# Where there is pressure, there is motivation? The impact of challenge-hindrance stressors on employees’ innovation performance

**DOI:** 10.3389/fpsyg.2022.1020764

**Published:** 2022-10-26

**Authors:** Guoqin Dou, Jinjuan Yang, Lifeng Yang, Bin Liu, Yunyun Yuan

**Affiliations:** ^1^School of Information Engineering, Fuyang Normal University, Fuyang, China; ^2^School of Economics, Fuyang Normal University, Fuyang, China; ^3^School of Business, Fuyang Normal University, Fuyang, China; ^4^School of Management and Economics, Beijing Institute of Technology, Beijing, China

**Keywords:** challenge stressors, hindrance stressors, innovation performance, emotional atmosphere, organizational climate

## Abstract

Based on the conservation of resource theory, this manuscript explores the impact mechanism of the challenge and hindrance stressors on innovation performance, introduces emotional atmosphere as a mediation variable, and on this basis, it examines the moderating role of organizational climate on emotional atmosphere and innovation performance. A two-wave survey of 263 subordinates and 29 supervisors who come from multisource field offered support for our model. Results showed that challenge stressors have a positive effect on innovation performance, positive emotional atmosphere mediates the relationship between challenge stressors and innovation performance; hindrance stressors have a negative effect on innovation performance, and negative emotional atmosphere mediates the relationship between hindrance stressors and innovation performance. Organizational climate strengthens the positive relationship between positive emotional atmosphere and innovation performance and weakens the negative relationship between negative emotional atmosphere and innovation performance. This study enriches the existing literature by identifying the impact of stressors on employee innovation performance and has certain practical significance for optimizing the management of enterprises and improving employee innovation performance.

## Introduction

Innovation, as the most important source for companies to improve their competitive advantage, is the cornerstone for companies to achieve sustainable development (Anderson and King, 1993; Camisón and Villar-López, 2014; Alfonso et al., 2022). How to effectively stimulate employees’ positive emotion and atmosphere, and improve the innovation performance of enterprises is a theoretical and practical problem to promote the high-quality development of enterprises in the era of knowledge economy (De Clercq and Pereira, 2020). However, although companies encourage and support employee innovation, the innovation performance of individuals, and even of companies, remains unsatisfactory. What makes it so difficult for employees to improve their innovation performance? Research has found that an improvement in employee innovation performance is not only be driven by the external incentives of the company (Baer et al., 2003), but is also dependent on the internal psychological motivation of employees. That is, only employees initiatively transform the perceived external pressure into the actual innovation action, such as efforts to create, introduce and apply new ideas, will they finally present innovative results and enhance innovation performance (Janssen and Van Yperen, 2004; Teabrat et al., 2019). The fierce global competition forces the members of the organization to actively deal with the external pressure sources, so as to maintain the internal creativity and external competitiveness of the organization (Trisakhon and Jermsittiparsert, 2019). At the same time, with the increasing competition in the job market and industry, external pressure sources affect the individual’s choice of innovation behavior, and promote their continuous innovation (Lambert and Hogan, 2010; Leong and Rasli, 2014), and become the endogenous power of organizational innovation. With enterprises encouraging and expecting employees to break through the conventional thinking and improve innovation performance situation, employees are facing more and more internal and external pressures. Therefore, the first objective of this study is to explore the mechanism and boundary conditions between individual stressors and innovation performance.

As one of the characteristics of the organization, work stress will affect the psychological state of employees and cause them to behave differently from the non-stress situation (Joseph and Ryan, 2019). Based on the research of Cavanaugh et al. (2000) on work stressors of managers, work stressors are divided into challenge and hindrance stressors. Challenge stressors refer to the stressors that employees consider useful with respect to work experience to create opportunities for personal growth (Baka and Prusik, 2021). A hindrance stressor is a stressor that interferes with or hinders an individual’s ability to achieve valuable goals. Some studies have shown that challenge stressors are positively correlated with employee work performance, while hindrance stressors are negatively correlated with work performance (Cavanaugh et al., 2000; Feifei and Jinghuan, 2015; Joseph and Ryan, 2019). Therefore, stressors have received widespread attention from academic circles. Different external stressors are not only affected innovation performance, but also affect the internal atmosphere of the organization (Tong et al., 2021). Emotional atmosphere, which is formed by the attitudes, experiences, and corresponding behavioral responses of each member of the organization to objective things, is a kind of emotional interaction or emotional integration (Hofmann and Stokburger-Sauer, 2017). Challenge stressors and hindrance stressors may bring different kinds of emotional atmosphere (positive emotional atmosphere and negative emotional atmosphere), which will become an important factor affecting employees’ innovation performance (Rostami et al., 2019). Therefore, in the face of “innovation requirements,” emotional atmosphere has become an inevitable research factor. The research shows that positive emotional atmosphere can improve individual innovation performance, while negative emotional atmosphere can hinder individual innovation performance (Joseph and Ryan, 2019). But when emotional atmosphere is used as an intermediate variable, what is the effect of the stressors and innovation performance? Worthy of attention for future research. Therefore, the second purpose of this study is to explore the deep mechanism between challenge-hindrance stressors and innovation performance by exploring the mediating effect of emotional atmosphere on stressors and innovation performance.

Furthermore, the conservation of resource theory assumes that people are always actively trying to maintain, protect and construct the valuable resources they think. The high level of organizational climate within an organization is a kind of high-quality resources that the organization gives to its members (Kassia et al., 2022), which is conducive to the development of work. It can significantly affect employees’ individual emotion and information processing ability, and then has a boundary regulating effect on the relationship between stressors and innovation performance (Pradoto et al., 2022). Organizational climate refers to the shared views and additional meanings of policies, practices, procedures and employee experience and the overall atmosphere perception of employ eyes’ experience and observation of getting reward, support and expected behavior. Research shows that team members with high organizational climate can effectively use social resources to explain and integrate different ideas and views (Kassia et al., 2022). Employees are full of confidence and trust in their innovation ability and can actively deal with challenge and hindrance stressors to form a good organizational climate, which plays an important role in improving innovation performance (Bu et al., 2021). Thus, this article drew on previous research results, combined with emotional atmosphere, to explore the establishment of challenge and hindrance stressors that influence innovation performance, and explored the conditions under which challenge-hindrance stressors exert the greatest effect to improve employee innovation performance. Emotional atmosphere, which is formed by the attitudes, experiences, and corresponding behavioral responses of each member of the organization to objective things, is a kind of emotional interaction or emotional integration (Hofmann and Stokburger-Sauer, 2017). When employees’ emotions are uncoordinated, they will have a negative impact on their work-life balance (Laine et al., 2020). According to research, a positive emotional atmosphere is conducive to the improvement of employees’ individual innovation performance and a negative emotional atmosphere has a hindering effect on employees’ individual innovation performance. Organizational climate refers to the shared views and additional meanings of policies, practices, procedures, and employee experience and their observed behaviors for rewards, support, and expectations (Benjamin and Reichers, 1983; Ostroff et al., 2003). Studies have shown that organizational climate affects the processing of employee emotions and information, which is very important for improvement in innovation performance (Chatzi et al., 2022). At the same time, different degrees of conformity between leadership style and organizational climate will also affect the improvement in innovation performance (Haakonsson et al., 2008; Chen, 2022). But when emotional atmosphere is used as an intermediate variable, what is the innovation performance of employees of different genders? What is the effect of the pressure source and innovation performance? In past research, few people have produced satisfactory answers. Thus, this article drew on previous research results, combined with emotional atmosphere, to explore the establishment of challenge and hindrance stressors that influence innovation performance, and explored the conditions under which challenge, and hindrance stressors exert the greatest effect to improve employee innovation performance.

In summary, we will examine the relationship between challenge stressors, hindrance stressors and innovation performance from the following aspects, as well as the role of emotional atmosphere and organizational climate in the above mechanism: Frist, analyze the impact mechanism of challenge and hindrance stressors on innovation performance; Second, incorporate the emotional atmosphere and organizational climate into the research framework, discuss the differences between the above two stressors and clarify the differential impact on the relationship between challenge and hindrance stressors and innovation performance. Finally, we collected corporate employee data through online questionnaires, used correlation and regression analyses and other methods to analyze the mechanism of challenge-hindrance stressors, and innovation performance, and provided correct guidance for improvement of employee innovation performance.

## Theory and hypotheses

### Challenge-hindrance stressors and innovation performance

Work stress refers to all types of pressures that are perceived by employees to significantly affect the development of an individual’s career in a work setting (Joseph and Ryan, 2019). McGarth (1987) believes that stress is the result of unmet needs, and due to their ability to cope with stress, different individuals have different stress experiences when individuals are in a state of imbalance between demand and capacity. Nowadays, emotional exhaustion, job burnout and job performance are regarded as the results of job stress (Kern et al., 2021). For example, research has found that when employees’ work stress is not properly recognized and handled, it may lead to emotional exhaustion and other problems (Pines and Maslach, 1978; Kern et al., 2021). Cavanaugh et al. (2000) divided job stressors into challenge and hindrance stressors based on the role of job stressors. From the perspectives of social cognition, resource conservation, social exchange, and activation theory, previous empirical studies have found that challenging stressors can improve individual self-efficacy (Bu et al., 2021), organizational support (Joseph and Ryan, 2019), organizational commitment (Montani et al., 2017), and job pro122sperity (Haldorai et al., 2022), and promote individuals to show positive innovative behavior (Joseph and Ryan, 2019; Tong et al., 2021). Employees’ individual challenge stressors are the foundation of organization and company performance, and the endogenous driving force of innovation.

Challenge stressors can promote the generation of innovative ideas and behaviors of employees, and then improve employees’ individual innovation performance (Joseph and Ryan, 2019), that is, challenge stressors have a positive influence on innovation performance (Noefer et al., 2009; Lee, 2011). First, challenge stressors force individuals to work at a high level of engagement. At this time, individuals are more curious and willing to engage in high-risk work activities (i.e., exploration) to solve work problems, thus having a positive impact on their innovation performance. Secondly, challenge stressors put forward demands for innovative problem solving and activate innovative thinking, and encourage employees to practice innovative ideas and behaviors, which is conducive to the improvement of innovation performance (Noefer et al., 2009). Research shows that employees under certain challenge stressor complete task performance and innovation performance through innovative work processes (West, 2002). Finally, challenge stressors give employees the expectation of future benefits: as long as they are able to cope with challenges, they will be able to achieve higher work performance, gain richer work experience and better work skills. This kind of expectation can motivate employees, offset the possible negative impact caused by high challenging stressors, and achieve higher level of performance results (Lepine et al., 2004; Zhang et al., 2015). Taken together, we propose the following hypothesis:

Hypothesis 1a: Challenge stressors positively affect innovation performance.

Hindrance stressors refer to the job requirements that interfere or hinder employees to achieve their own values and goals (Rodell and Judge, 2009). Hindrance stressors have a negative effect on innovation performance, and its emotional and gradual blocking characteristic cannot be ignored (Joseph and Ryan, 2019). Regardless of the extent of hindrance stressors, it will cause individual resource imbalance, and then lead to job burnout, emotional exhaustion, feedback avoidance, and performance deficiencies and other problems (Baka and Prusik, 2021). The emotionality of blocking stressors pointed out, due to the time accumulation effect of employees’ perception of external things, when individuals’ pressure gradually increases and produces negative effects, the emotional nature of hindrance stressors (Ramos and Mormède, 1998) will lead to negative work emotions such as job burnout, which will affect the completion of individual innovation work and innovation performance (Ordóñez et al., 2016). At the same time, when an individual employee realizes that he/she cannot cope with work needs regardless of the effort, strong hindrance stressors force the individual to spend a lot of internal resources for emotional regulation, this excessive emotion regulation leads to the imbalance of individual resources, and then produces adverse results such as emotional exhaustion (Brotheridge and Lee, 2002; Stphane, 2005), leading to the decline of individual innovation performance. Lin et al. (2015) studied the relationship between work stressors and performance of employees from the perspective of personal resources (i.e., sense of responsibility), showed that the increase of hindrance stressors will lead to the decrease of task performance and innovation performance. Finally, hindrance stressors put individual employees in a state of high stress, making the individual’s attention too concentrated, thereby weakening their judgment, increasing the probability of making mistakes, as a result, employees are unable to improve work efficiency and innovation performance (Jamal, 1984; Seipp, 1991). Individuals believe that they lack sufficient resources to cope with work needs and will give up self-effort to actively meet work needs, under high work pressure subjectively, thus affecting individual and team performance. Taken together, we propose the following hypothesis:

Hypothesis 1b: Hindrance stressors negatively affect innovation performance.

### The mediating role of emotional atmosphere

Emotional atmosphere refers to a kind of emotional interaction or integration formed by individual factors, such as individual emotions and emotional characteristics of each member in an organization through shared experiences and implicit or explicit sharing processes (Susana and Itziar, 2007; Michael and Thomas, 2019). Challenge stressors positively affect employees’ individual positive emotional atmosphere (Jiang et al., 2020). First, challenge stressors have a potential role in promoting employees’ personal growth, self-efficacy, stimulating personal sense of achievement and positive emotion perception (Mohammadreza et al., 2020). For example, challenge stressors positively stimulate individual growth, learning and goal achievement, which provides information about the progress of some valuable results, and positive responses to valuable events, so as to improve individual physiological function and subjective pleasure (Watson et al., 1988), and then trigger positive emotional atmosphere within the organization (Lazarus, 1991; Cavanaugh et al., 2000). Second, the challenging stressors are helpful for individuals to improve their income or promote their growth, so they can stimulate positive feelings of individuals and urge them to adopt proactive or problem solving oriented coping strategies. This positive attitude toward challenge stressors not only enables individuals to have positive emotions (Joseph and Ryan, 2019), but also spreads such positive emotions within the organization, which is conducive to the formation of an overall positive emotional atmosphere within the organization (Sun et al., 2021). Finally, challenging stressors help to stimulate individuals’ perception of work emotions, such as concentration and time pressure. Such positive emotions make individuals show concentration and determination, and then make individuals experience greater commitment and pleasure in work, which will stimulate individuals’ positive emotions (Kern et al., 2021). Ultimately, it is conducive to the formation of positive emotional atmosphere within the organization.

In addition, Positive emotional atmosphere also has positive effect on employees’ innovation performance. Compared with the negative and neutral mood, the individuals in the positive emotional atmosphere have more outstanding creativity and higher innovation performance For example, positive emotional atmosphere causes employees to have a stronger task focus (Barsade, 2002). Employees exhibit better work performance and creativity in solving creative problems (Grawitch et al., 2003) and are more likely to increase employees’ perception of task performance and innovation performance (Joseph and Ryan, 2019). What’s more, under the positive atmosphere, the mutual cooperation, communication and discussion among employees are more frequent, and there are more extended interactions within the organization (Methot et al., 2021). Such free, pleasant and positive interactions will stimulate employees’ creative thinking and ideas to the maximum extent (Methot et al., 2017), and thus positively affect innovation performance. Finally, the positive emotional atmosphere makes individuals confident in their innovation ability and encourages them to maintain high enthusiasm to explore new work methods and processes, so as to achieve high innovation performance (Rhee, 2006). Taken together, we propose the following hypothesis:

Hypothesis 2a: Positive emotional atmosphere plays a mediating role between challenge stressors and innovation performance.

On the other hand, the generation or improvement of hindrance stressors makes it difficult for employees to achieve their work goals, resulting in the enhancement of negative emotional atmosphere (Zhang et al., 2018). And then lead to the decrease of work performance and innovation performance (Yan et al., 2022). Moreover, hindrance stressors can also lead to resource imbalance, accelerate resource loss, lead to job burnout, emotional exhaustion and other problems, and lead to the enhancement of negative emotional atmosphere (Widmer et al., 2012), which makes it difficult for employees to improve innovation performance. At the same time, based on the conservation of resource theory, employees will have psychological anxiety under the influence of hindrance stressors, which will enhance the negative emotional atmosphere and reduce their innovation performance (Nathan et al., 2007). Secondly, hindrance stressors are stressors that can lead to the enhancement of negative emotions such as tension, higher negative mood atmosphere will reduce employee satisfaction (Joseph and Ryan, 2019), which may affect the creativity enthusiasm of employees, and have a negative impact on their innovation performance (Jackson and Schuler, 1985; Nathan et al., 2007). Finally, hindrance stressors will lead employees to enter a state of psychological stress (Tong et al., 2021), which enhances the negative emotional atmosphere (Sonnentag and Fritz, 2015; Zhang et al., 2015). However, this negative emotional atmosphere will break the work-life balance of employees, makes it difficult for them to meet the performance requirements required by their work, which will lead to the reduction of individual work performance and innovation performance (Weiss and Cropanzano, 1996; Niebusch and Moran, 2019). In summary, the following hypothesis is proposed:

Hypothesis 2b: Negative emotional atmosphere plays a mediating role between hindrance stressors and innovation performance.

### The moderating role of organizational climate

Organizational climate refers to an overall attribute of an organization, which is the enduring characteristic of the organization or environment perceived by the organization or its members (Forehand and Haller, 1964; Schneider and Bartlett, 2010). Based on the conservation of resource theory, positive organizational climate is a valuable social resource that has a significant impact on employees’ individual innovation performance (Contreras et al., 2021). On the one hand, research have shown that, team members with positive organizational climate can effectively use social resources to explain and integrate different ideas and views, so they are more focused on creative tasks and less distracted by psychological anxiety, enhance the positive emotional atmosphere (Bu et al., 2021). At this time, the increase of self-owned resources will be generated by employees, which will improve the probability of employees obtaining the value-added spiral (Grawitch et al., 2009). Thus, the possibility of falling into the spiral of resource loss is reduced, and the negative results such as job burnout caused by resource loss are alleviated, which has a significant positive impact on the creativity and innovation performance of employees (Pritchard and Karasick, 1973; Anthonia, 2011). Organizational climate is instrumental in the three interactions among leaders, employees, and the environment, enhances employees’ psychological perception of the organization, help employees take a more positive attitude to deal with difficulties at work, which is more conducive to the completion of employees’ initiative innovation behavior and the improvement of innovation performance (Spillane, 2005). Good organizational climate guides employees to pay close attention to positive results of events, encourages employees to implement positive actions and enhances positive emotional atmosphere (Marius et al., 2020), enable employees to generate more innovative ideas, and improve individual performance (Burns et al., 1978; Ghafoor et al., 2011). On the other hand, research found that team members with negative organizational climate will hold the view that it is difficult to replenish or return the existing resource investment, so the motivation to continue to invest will be reduced accordingly, which will lead to employees’ individual negative work attitude and performance behavior (Pradoto et al., 2022), and will lead to a significant reduction in employees’ innovation performance. Therefore, organizational climate will affect the implementation of individual innovative ideas and the results of innovative behavior.

Positive organizational climate helps employees to resolve the stress and burnout caused by the loss of resources (Methot et al., 2021). In this situation, the high organizational climate enables employees to obtain more support resources from the organization, establish commitment and trust in the organization, and then reduce the possibility of job burnout and turnover, make individuals take a positive attitude to deal with the difficulties in the innovation process, thereby enhancing their innovative performance (Sun et al., 2021). Secondly, the conservation of resource theory holds that the higher the organizational climate, the more original psychological resources the individual employee has, and the stronger the pressure relief ability (Laeeque et al., 2022), help individuals better adjust the relationship between emotional atmosphere and innovation performance, and the positive effect of positive emotional atmosphere on innovation performance increases (Joseph and Ryan, 2019). Finally, the high organizational climate is conducive to inject the motivation of “positive work” into employees, stimulate their positive emotion and emotional commitment to the organization, help establish a high positive emotional atmosphere within the company, and then help employees achieve high innovation performance. On the contrary, if the organizational climate is low, employees will not feel organizational support, which will cause resource imbalance, weaken the effectiveness of positive emotional atmosphere, and then lead to insufficient innovation performance. In summary, the following hypothesis is proposed:

Hypothesis 3a: Organizational climate positively moderates the relationship between positive emotional atmosphere and innovation performance; that is, the higher the organizational climate, the higher the positive impact of positive emotional atmosphere on innovation performance.

Organizational climate can significantly regulate the relationship between negative emotional atmosphere and innovation performance and weaken the negative effect of negative emotional atmosphere on innovation performance. Firstly, positive organizational climate can give individual employees organizational support, improve the resource imbalance in the working environment, enhance individual employees’ high commitment and high attachment to the enterprise, reduce the negative impact of negative emotional atmosphere on innovation performance, and then improve innovation performance; Secondly, positive organizational climate can reduce the impact of negative emotional atmosphere, strengthen the role of positive emotional atmosphere, stimulate employees’ innovative behavior, and then improve innovation performance (Scott and Bruce, 1994). On the contrary, negative organizational climate will lead to individual psychological pressure, destroy the balance of individual resources, and then lead to emotional exhaustion and burnout (Bai et al., 2021), which may strengthen the negative impact of negative emotional atmosphere on innovation performance, this leads to employees’ behavior of recovering and preserving potential value at work, and reducing innovation performance. At the same time, negative organizational climate will also make employees feel depressed and enhance the negative emotional atmosphere, which count against the exertion of employees’ initiative and the improvement of innovation performance. In summary, the following hypothesis is proposed:

Hypothesis 3b: Organizational climate negatively moderates the relationship between negative emotional atmosphere and innovation performance; that is, the higher the organizational climate, the weaker the positive impact of negative emotional atmosphere on innovation performance.

**FIGURE 1:**
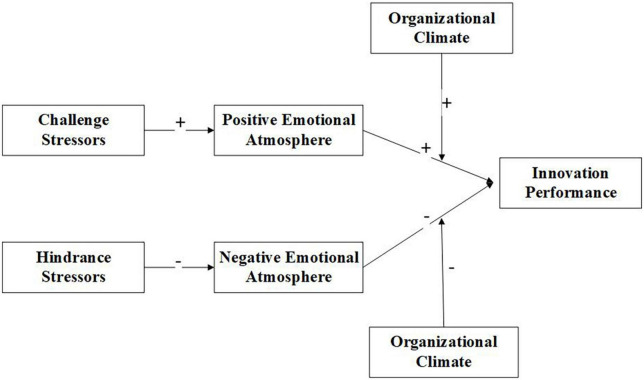
Theoretical model.

In accordance with the literature review and the hypotheses outlined above, our research framework is illustrated in Figure 1.

## Materials and methods

### Participant and procedures

The scope of this article was mainly focused on Guangdong Province, Gansu Province, and involved the financial, manufacturing, and service industries. The selected enterprises had been established for more than 2 years, and the scale of enterprises had reached more than 20 people. All employees participating in the questionnaire survey had volunteered and anonymity was assured. The specific operating procedures were as follows: Firstly, five enterprises were selected considering various factors such as industry, difficulty of investigation and number of employees, then we find a contact person within each company, explain to them the purpose, content, and procedures of the investigation to increase their trust toward the research. Secondly, after we received support from the human resource managers of each company, a cover letter was distributed to each participant that provided information about the purposes of the research. Third, to ensure that the participants were able to complete the questionnaire carefully and thoroughly, we requested each contact to use an on-site distribution and recycling method, and we safeguarded the anonymity and confidentiality of all the retrieved questionnaires. Finally, considering the reliability and validity of the questionnaire and the common method bias, this study designed two-wave time-delay paired data to test the theoretical hypothesis.

As such, at Stage 1, questionnaires were distributed to 400 employees, and employees answered questions about independent variables (challenge stressors and hindrance stressors), mediators (i.e., positive emotional atmosphere and negative emotional atmosphere), moderator (i.e., organizational climate), and control variables. After 4 weeks, we received feedback and obtained 334 valid questionnaires, giving a response rate of 83.5%. At Stage 2, questionnaires were distributed to 35 supervisors to rate their subordinates’ innovation performance and only six supervisors failed to complete their questionnaires, giving a response rate of 82.9%. After sorting out and analyzing the collected questionnaires and eliminating incomplete or inconsistent questionnaires, a total of 263 valid questionnaires were retrieved, with a response rate of 65.75%. The demographic data were as follows: Approximately 59.7% of the participants were males. The 26–35-year-old age group was the largest (55.5%), followed by the 25-year-old (27.4%). 38.4% had worked with their superiors for 1∼3 years, 20.5% had worked with their superiors for more than 6 years. Most of the participants had a tertiary education, 11.8% had a master’s degree, and 56.7% had been awarded a bachelor’s degree.

### Measures

The measures used in this study were adapted from existing validated scale, and all measurement items were translated from English into Chinese using a back-translation procedure (Brislin, 1980). All were rated on a scale from 1 (strongly disagree) to 5 (strongly agree).

### Challenge and hindrance stressors

Using the 8-item scale compiled by Rodell and Judge (2009) the scale was divided into two dimensions (challenge and hindrance stressors) to reflect the individual employee’s perception of two different sources of stress at work. Representative items, such as “Today, my job has required me to use a number of complex or high-level skills,” “Today, I have experienced severe time pressures in my work,” and “Today, I have had many hassles to go through to get projects/assignments done.” The mean coefficient alpha (across days) was 0.704 for challenge stressors and 0.71 for hindrance stressors.

### Innovation performance

Using the innovation performance scale in the role-based performance scale compiled by Janssen and Van Yperen (2004), the scale was divided into three dimensions (creativity generation, creativity promotion, and creativity realization). The role-based performance scale contained a total of nine items, such as “Creating new ideas for difficult issues (idea generation),” “Acquiring approval for innovative ideas (idea promotion),” and “Transforming innovative ideas into useful applications (idea realization).” The mean coefficient alpha (across days) was 0.911.

### Emotional atmosphere

Using the emotional atmosphere scale compiled by Watson et al. (1988), the scale was divided into two dimensions (positive emotion and negative emotional atmosphere). The scale has 16 items, such as “excitement,” “happy,” “fear,” and “impatient.” The mean coefficient alpha (across days) was 0.928 for positive emotional atmosphere and 0.930 for negative emotional atmosphere.

### Organizational climate

Using the organizational climate scale compiled by Tierney and Farmer (2002), the scale consisted of 3 dimensions and a total of 11 items, such as “Our organization members cooperate with each other tacitly,” “My organization encourages employees to actively innovate,” and “Organizational distribution so the task for everyone is fair.” The mean coefficient alpha (across days) was 0.924.

### Control variables

Considering the research object was individual employees, in order to exclude the influence of demographic variables on the research results, combined with previous studies, four control variables (gender, age, education background, and working years) were selected.

## Results

### Measurement model

We conducted Confirmatory Factor Analysis (CFA) to assess the discriminant validity of the variables used in the study, in which all indicators were loaded onto their respective latent variables (i.e., Challenge stressors, Hindrance stressors, Positive emotional atmosphere, Negative emotional atmosphere, Organizational Climate, Innovation performance). As indicated in Table 1, the six-factor model shows a relatively good fit with the data (χ^2^ = 1546.335 df = 887, χ^2^/df = 1.743 < 2, CFI = 0.908, TLI = 0.902, IFI = 0.909, RMSEA = 0.053 < 0.06), and all standardized factor loadings were significant at the *p* < 0.001 level. Moreover, all the alternative models had a significantly worse fit than the hypothesized six-factor model (all Δχ^2^ tests, *p* < 0.001) and showed a less desirable model fit (all CFIs < 0.90). The six-factor model was retained for hypothesis testing.

**TABLE 1:** Confirmatory factor analysis results.

Model	χ^2^	DF	χ^2^/df	RMSEA	IFI	TLI	CFI
One factor	4294.273	902	4.761	0.12	0.532	0.506	0.529
Two factors	4051.544	901	4.497	0.116	0.565	0.541	0.562
Three factors	3969.624	899	4.416	0.114	0.576	0.551	0.574
Four factors	2496.303	896	2.786	0.083	0.779	0.765	0.778
Five factors	2350.615	892	2.635	0.079	0.799	0.785	0.797
Six factors	1546.335	887	1.743	0.053	0.909	0.902	0.908

### Descriptive statistical analysis

This study used SPSS 26.0 to perform descriptive statistics and analysis of the research data. The results of statistical analysis represented information about challenging stressors, hindrance stressors, positive emotional atmosphere, negative emotional atmosphere, organizational climate, and innovation performance, including the mean value of variables and correlation coefficient, are shown in the Table 2.

**TABLE 2:** Means, standard deviations, and correlations among variables.

Variable	M	SD	1	2	3	4	5	6
1. CS	3.6977	0.59609	**(0.704)**					
2. HS	3.423	0.64019	–0.034	**(0.710)**				
3. PEA	3.5862	0.70403	0.146*	−0.427**	**(0.911)**			
4. NEA	3.4292	0.78261	0.231**	0.568**	−0.505**	**(0**.**928)**		
5. OC	3.8287	0.7165	0.054	–0.076	0.336**	–0.179**	**(0.930)**	
6. IP	3.5138	0.75372	0.262**	−0.419**	0.547**	–0.444**	0.267**	**(0.924)**

N = 263. *p < 0.05, **p < 0.01.

CS, Challenge stressors; HS, Hindrance stressors; PEA, Positive emotional atmosphere; NEA, Negative emotional atmosphere; OC, Organizational Climate; IP, Innovation performance. Scale reliabilities (Coefficient alpha) are on the diagonal.

According to Table 2, challenge stressors were significantly positively correlated with positive emotional atmosphere, organizational climate, and innovation performance. Hindrance stressors were negatively correlated with innovation performance, and positively correlated with negative emotional atmosphere and organizational climate. The correlation analysis of the scale initially verified the relevant research hypothesis of this study.

### Hypothesis testing

This study used the hierarchical regression method to analyze the mediation effect, the analysis of which is shown in Table 3. Employee gender had a significant impact on innovation performance, while age, education, and working years had a relatively low impact on innovation performance. Challenge stressors had a positive correlation with innovation performance (model 1, *r* = 0.268, *p* < 0.01). Hypothesis H1a was verified. Challenge stressors positively affected the positive emotional atmosphere (model 2, *r* = 0.173, *p* < 0.01), and positive emotional atmosphere positively affected innovation performance (model 3, *r* = 0.467, *p* < 0.01), hypothesis H2a was supported. Finally, after adding an intermediary variable (positive emotional atmosphere) between challenge stressors and innovation performance, it was shown that the impact coefficient of challenge stressors on innovation performance became smaller. But it still has a significant impact (model 4, *r*_1_ = 0.223, *p* < 0.01; *r*_2_ = 0.48, *p* < 0.01). Thus, positive emotional atmosphere plays a mediating role between challenge stressors and innovation performance, and hypotheses H2a were verified.

**TABLE 3:** Results of mediation of positive emotional atmosphere in the relationship between challenge stressor and innovation performance.

Variables	Model 1	Model 2	Model 3	Model 4
	IP	PEA	IP	IP
Gender	−0.547**	−0.587**	−0.38**	−0.266**
Age	0.081	−0.075**	0.152	0.117
Education	0.086	0.191	–0.01	–0.005
Working years	0.003	0.127**	–0.071	–0.058
CS	0.268**	0.173*		0.223**
PEA			0.467**	0.48**
r2	0.215**	0.263**	0.333**	0.363**
r2_a	0.2	0.249	0.32	0.348

N = 263. *p < 0.05, **p < 0.01.

CS, Challenge stressors; PEA, Positive emotional atmosphere; NEA, Negative emotional atmosphere; IP, Innovation performance.

It can be seen from Table 4 that hindrance stressors had a negative correlation with innovation performance (model 1, *r* = –0.359, *p* < 0.01), and hypothesis H1b was verified. Hindrance stressors had a significant positive correlation effect on negative emotional atmosphere (model 2, *r* = 0.595, *p* < 0.01), assuming that H2b had been initially verified. And negative emotional atmosphere negatively affected innovation performance (model 3, *r* = −0.344, *p* < 0.01), hypothesis H2b was supported. After adding an intermediary variable (negative emotional atmosphere) between hindrance stressors and innovation performance, it was shown that the impact coefficient of hindrance stressors on innovation performance became smaller. But it still has a significant impact (model 4, *r*_1_ = −0.215, *p* < 0.01; *r*_2_ = −0.242, *p* < 0.01). In summary, negative emotional atmosphere had a mediating effect on hindrance stressors and innovative performance.

**TABLE 4:** Results of mediation of negative emotional atmosphere in the relationship between hindrance stressor and innovation performance.

Variables	Model 1	Model 2	Model 3	Model 4
	IP	PEA	IP	IP
Gender	−0.427**	0.283**	−0.538**	−0.358**
Age	0.059	0.19*	0.172	0.105
Education	0.05	−0.148*	0.024	0.014
Working years	0.003	−0.128**	–0.047	–0.028
HS	−0.359**	0.595**		−0.215**
NEA			−0.344**	−0.242**
r2	0.25**	0.38**	0.268**	0.29**
r2_a	0.236	0.368	0.254	0.273

N = 263. *p < 0.05, **p < 0.01.

HS, Hindrance stressors; PEA, Positive emotional atmosphere; NEA, Negative emotional atmosphere; IP, Innovation performance.

To better analyze the mediating effect of positive and negative emotional atmosphere, we further applied the Sobel test and bootstrap samples analysis; the results are shown in Table 5. Positive emotional atmosphere had a significant mediating effect on challenge stressors and innovation performance. Negative emotional atmosphere had a significant mediating effect on hindrance stressors and innovation performance. Therefore, hypotheses H2a, H2b were verified.

**TABLE 5:** Tests on the mediation effect of positive and negative emotional atmosphere.

Stressors	Intermediary path	Innovation performance	Total effect	Direct effect	Indirect effect
CS	PEA	IP	0.3317**	0.2354**	0.0963*
HS	NEA	IP	−0.4931**	−0.2901**	−0.203**

N = 263. *p < 0.05, **p < 0.01.

CS, Challenge stressors; HS, Hindrance stressors; PEA, Positive emotional atmosphere; NEA, Negative emotional atmosphere; IP, Innovation performance.

Finally, we test the moderating effect of organizational climate on the relationship between positive emotional atmosphere and innovation performance, and between negative emotional atmosphere and innovation performance, and before proceeding, we centralization the related variables. As shown in Table 6, positive emotional atmosphere had a significant positive correlation with innovation performance (model 1, *r* = 0.467, *p* < 0.01), and organizational climate had a significant positive correlation with innovation performance (model 2, *r* = 0.111, *p* < 0.01). On this basis, the interaction item of positive emotional atmosphere and organizational climate was added, and it was shown that organizational climate had a positive role in regulating positive emotional atmosphere and innovation performance (model 3, *r*_3_ = 0.381, *p* < 0.01), assuming H3a was verified., the interaction effect is significant. Negative emotional atmosphere had a significant negative correlation effect on innovation performance (model 4, *r* = −0.344, *p* < 0.01). On this basis, the interaction item of negative emotional atmosphere and organizational climate was added, and it was shown that organizational climate had a significant positive regulatory effect on negative emotional atmosphere and innovation performance (model 6, *r* = 0.281, *p* < 0.01). Hypothesis H3b was verified. In summary, organizational climate played a moderating role among positive emotional atmosphere, negative emotional atmosphere, and innovation performance.

**TABLE 6:** Analysis results of the moderating effect of organizational climate.

Variables	Model 1	Model 2	Model 3	Model 4	Model 5	Model 6
	IP	IP	IP	IP	IP	IP
Gender	−0.38**	−0.396**	−0.345**	−0.538**	−0.526**	−0.528**
Age	0.152	0.168	0.138	0.172	0.201	0.186
Education	–0.01	–0.03	–0.02	0.024	–0.021	–0.011
Working years	–0.071	–0.08	–0.065	–0.047	–0.069	–0.065
PEA	0.467**	0.429**	−0.591*			
NEA				−0.344**	−0.319**	−1.169**
OC		0.111*	−0.883**		0.189**	−0.509*
OC*PEA			0.381**			
OC*NEA						0.281**
r2	0.333**	0.344*	0.388	0.268**	0.302**	0.321**
r2_a	0.32	0.328	0.371	0.254	0.286	0.302

N = 263. *p < 0.05, **p < 0.01.

PEA, Positive emotional atmosphere; NEA, Negative emotional atmosphere; OC, Organizational Climate; IP, Innovation performance.

**FIGURE 2:**
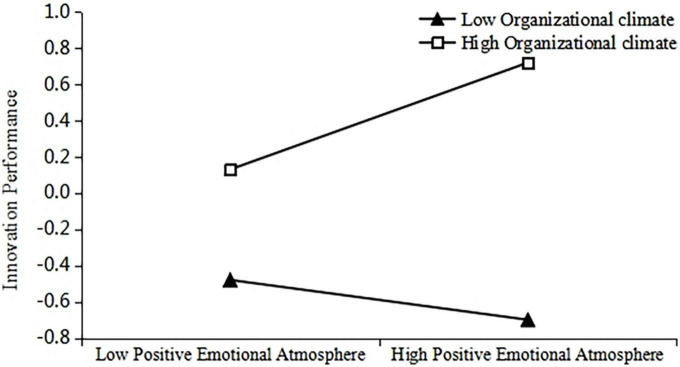
The moderating role of organizational climate on the relationship between positive emotional atmosphere and innovation performance.

**FIGURE 3:**
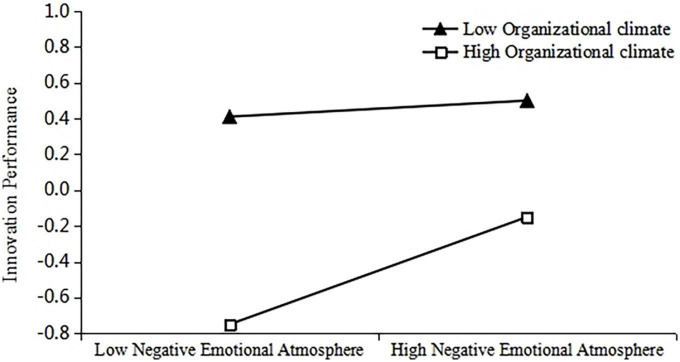
The moderating role of organizational climate on the relationship between negative emotional atmosphere and innovation performance.

To further test the moderating effect of organizational climate, a diagram depicting the moderating effects of organizational climate on innovation performance by regulating positive and negative emotional atmosphere was developed (Figures 2, 3). As can be seen from the slope of the middle line segment in Figures 2, 3, when the organizational climate was high, the positive influence of positive emotional atmosphere on innovation performance was higher than the low organizational climate, and the negative influence of negative emotional atmosphere on innovation performance was lower than the low organizational climate. H3a and H3b were thus verified.

## Discussion

High-intensity, high-speed global competition and the uncertainty of the external business environment have directly led to increased pressure on employees (Samrong, 2018; Hailun, 2022; Pradoto et al., 2022). In this context, how to actively guide employees, adjust challenging-hindrance stressors is the key to improving employee innovation performance (Webster et al., 2010; Joseph and Ryan, 2019). In view of this, we constructed a challenge-hindrance stressors—emotional atmosphere—innovation performance theoretical model to determine the innovation performance of employees and analyzed the mediating effect of emotional atmosphere and the regulatory role of the organizational climate. The results showed that challenge stressors had a positive impact on employees’ innovation performance, and hindrance stressors had a negative impact on employees’ innovation performance. Positive emotional atmosphere was positively correlated with innovation performance, and negative emotional atmosphere was negatively correlated with innovation performance, which is consistent with previous research (Gasper, 2003; Isgett and Fredrickson, 2004). At the same time, the emotional atmosphere played a mediating role between challenge and hindrance stressors and innovation performance. Organizational climate significantly regulated the relationship between emotional atmosphere and innovation performance. When organizational climate is positive, positive emotional atmosphere had a more significant positive impact on innovation performance and negative emotional atmosphere had a reduced negative impact on innovation performance. These conclusions provide theoretical guidance for companies to encourage employees to improve their innovation performance.

### Theoretical implications

From the perspective of conservation of resource theory, we clarify the relationship between challenge-hindrance stressors and innovation performance. Research have confirmed that there is a close relationship between stress and individual innovation performance (Joseph and Ryan, 2019), but inconsistent conclusions have been drawn on the specific influence mechanism between the two. Among them, there has not reached a consensus on the relationship between challenge stressors and innovation performance. However, we have reached a consensus on the negative impact of hindrance stressors on innovation performance (Baer et al., 2003; Ohly et al., 2006; Lin et al., 2015). Therefore, we set off from the conservation of resource theory, taking challenge stressors as a positive situation feature and hindrance stressors as a negative situation feature (Baka and Prusik, 2021; Tong et al., 2021). At the same time, positive emotional atmosphere and negative emotional atmosphere were used as mediating variables to study the effects of challenge-hindrance stressors on innovation performance (Kopelman et al., 1990; Hofmann and Stokburger-Sauer, 2017). Employees’ self-regulation of challenge and hindrance stressors to innovative work processes and methods can enhance the positive emotional atmosphere, weaken the negative emotional atmosphere, and thus improving individual innovation performance. This research enriches the research on innovation performance, effectively expands the research on stressors, and helps to better understand the specific ways that challenge, and hindrance stressors affect innovation performance.

Secondly, our research results indicated that emotional atmosphere mediates the relationship between challenge-hindrance stressors and innovation performance, while positive emotional atmosphere mediate the positive relationship between challenge stressors and innovation performance, negative emotional atmosphere mediate the negative relationship between hindrance stressors and innovation performance. Previous studies on the mechanism of challenge-hindrance stressors and innovation performance have mostly discussed from the perspectives of organizational support, self-efficacy, leadership-subordinate relationship, active and passive behaviors (Tong et al., 2021; Haldorai et al., 2022). However, little attention has been paid to how the internal emotional atmosphere of an organization activates or inhibits employees’ innovation performance under stressful situations. In view of the important influence of emotion on individual behavior, it is especially valuable to help employees understand the internal mechanism between external stressors and innovation performance (Ali et al., 2018; Joseph and Ryan, 2019). From the perspective of emotional atmosphere, this study explores the mediating role of positive and negative emotional atmosphere between challenge-hindrance stressors and innovation performance, thus, improving the research on the mechanism of challenge-hindrance stressors to innovation performance to a certain extent.

Finally, the research shows that organizational climate positively regulates the relationship between positive emotional atmosphere and innovation performance, and negatively regulates the relationship between negative emotional atmosphere and innovation performance (Havlovic and Keenan, 1995; Contreras et al., 2021). Specifically, employees with a positive organizational climate are likely to find key information or inspiration for innovation with positive emotions, and are more likely to show innovative behaviors, and then promote the improvement of innovation performance (Bu et al., 2021), when faced with challenging stressors (Binnewies and Wrnlein, 2011). In addition, the conservation of resource theory holds that, in a positive organizational climate, employees are more likely to actively acquire information and resources in interpersonal communication, stimulate innovative ideas, promote the generation of innovative behaviors, and then promote the improvement of innovation performance (Boswell et al., 2004). At the same time, in the face of hindrance stressors, employees with positive organizational climate are easy to adjust negative emotions and deal with job burnout. Positive organizational climate weakens the negative impact of negative emotional atmosphere on innovation performance, and then reduces the negative impact of hindrance stressors on innovation performance (Marius et al., 2020).

### Practical implications

To promote the sustainable development of the company and improve the innovation performance of employees, companies should take various measures to encourage employees to take innovative behaviors, so as to improving innovation performance (Rastegary and Landy, 1993; Rich et al., 2010; Sanger and Chienwattanasook, 2019). The relevant research results of this article have the following guiding significance for the management practice of enterprises. In the management practice, managers should pay more attention to employees’ challenge-hindrance stressors, reasonably set challenging tasks for employees, ensure sufficient task complexity and challenge expectations for employees. And at the same time, managers should not give too much pressure to employees, so as to prevent the challenging stressors change to hindrance stressors, which will bring negative emotional atmosphere and adversely affect innovation performance. In short, it is to play the maximum role of challenging stressors, and guard against the negative impact of hindrance stressors, encourage employees to constantly take innovative behaviors, and improve innovation performance (Devasheesh and Theresa, 2009).

Second, companies should show concern about the emotional atmosphere at work in their daily management activities (Chandaeng and Saisopa, 2018), while strengthening the role of positive emotional atmosphere to keep employees in a state of positive emotional atmosphere to the greatest extent (Schaubroeck et al., 2000). Positive emotional atmosphere plays an important role in building positive working atmosphere within an organization. Therefore, it is suggested that managers should establish the awareness of learning organization in the organization. Such awareness of learning and communication can keep employees in a positive emotional atmosphere most of the time (Neeama, 2022). Meanwhile, continuous awareness of learning and sharing can also help employees actively cope with negative emotions. What’s more, emotions are unpredictable (Sun et al., 2021), but it is very important that they need to be regulated. That’s why we suggest that managers establish an “emotional venting zone” in the organization to provide a private place for employees to releasing their negative emotions.

Finally, combined with the positive regulatory role of positive organizational climate between emotional atmosphere and innovation performance, managers must attach importance to the creation of positive organizational climate. Creating a good organizational climate is the key to improving innovation performance (Marius et al., 2020). Therefore, it is suggested that managers create an organizational climate where employees can safely share knowledge, information and creative ideas, so that employees can freely communicate and sharing, and thus having a positive impact on innovation performance. For example, the organization can set up a coffee area in the workplace, where employees could have some small talk interaction (Methot et al., 2021) and relax. Studies have shown that a relaxing environment is more conducive to creative ideas (Marius et al., 2020). Managers can also organize regular communication days within the group to encourage full communication among employees, shorten the psychological distance between employees, and enhance the trust between employees. The trust relationship can promote the full communication between employees, and then have a positive impact on innovative work (Yuan et al., 2021).

### Limitations and future research

Although this study uses empirical evidence to test the expected model, there are still some limitations. First, although we have used two-wave matching design to collect data, the design is cross-sectional and causality in the relationships could not be tested. Future research could adopt a longitudinal study with long-term investigation to consider the relationship between challenge-hindrance stressors and innovation performance. Second, this research was one-sided in the research on the internal mechanism of challenge and hindrance stressors, and innovation performance. Future research can use self-efficacy and self-loss as intermediary variables to further explore the internal mechanism of the impact of stressors on innovation performance. Finally, employees’ innovation performance is affected by a variety of positive and negative situational factors and presents different states. Based on the conservation of resource theory, previous research focused on emotional exhaustion and job burnout. However, there are few research on how to avoid the negative effects of stress. In the future, researcher can try to learn from the stress generation mechanism explained by COR theory and carry out the research on management strategy from three aspects: employee resource protection, acquisition, and utilization, so as to improve and develop the research on stress management.

## Data availability statement

The raw data supporting the conclusions of this article will be made available by the authors, without undue reservation.

## Author contributions

GD and JY: conceptualization and formal analysis. BL and YY: methodology, validation, and investigation. BL: software analysis and writing—original draft preparation. GD and LY: writing—review and editing. LY: supervision, project administration, and funding acquisition. All authors have read and agreed on the final version of the manuscript.
